# Medical Opinion and Movement

**Published:** 1908-09-26

**Authors:** 


### Medical Opinion and Movement.

Bingel has closely studied a series of forty cases of paratyphoid fever, and has recently published his results. He finds, in agreement with most authorities, that in the majority of cases the ordinary clinical signs of enteric fever—such as bronchitis, rose-coloured spots, enlarged spleen, leucopenia, and a positive Ehrlich's diazo reaction—usually fail or are only present in a very slight degree. The clinical picture of a case of paratyphoid is similar to that of an acute gastro-enteritis. Whether the disease is a real clinical entity or not cannot at present be determined, but the author is inclined to deny that it is such. According to him paratyphoid fever is essentially a gastro-enteritis, caused by a specific bacillus. It cannot be classed as a mild degree of enteric fever, and therefore the name "paratyphoid fever" should be given up, as the disease has really nothing in common with ordinary typhoid fever. These conclusions are not by any means new. English authorities have regarded paratyphoid fever, as a clinical entity, with well-grounded scepticism, but hitherto so little work has been done on this subject that one must agree with the author that the time has not yet arrived for a final decision to be made. The close study of his long series of cases, personally observed, justifies Bingel in his conclusions, but it is to be hoped that further research will be made, and the claims of paratyphoid to be considered as a definite disease fully threshed out in the near future.

---

It has been noted that in some cases of morphomania, and in some cases only, there is a curious development of bluish pigmentation in the skin in the form of spots; and from time to time the condition causes a difficulty in diagnosis when it is not known that the patient is a drug-taker by injection. The precise nature of the spots was for some time a mystery. It seems to have been clearly established that the blue spotting is not pathognomonic of morphine injecting, for a similar condition has been noted in a few cases after the injection of cocaine. It also seems to have been demonstrated with certainty that the blue pigment spots are not directly connected with either the morphine or the cocaine itself, but rather with the needle used in making the injections. Some of the affected skin has been excised and chemically analysed, with the result that the apparently blue pigment is found to be a form of reduced iron. The question at once arises, Why do some cases suffer from this blue tattooing, and others not? Apparently it is a question of the condition of the iron or steel needle used in making the injections and of the state of the cocaine or morphine solution into which this iron or steel needle is being repeatedly dipped. If an experimental injection is made with a solution of cocaine or morphine hydrochloride containing a yellow precipitate of iron, an iron needle being used for the injection, a blue stain appears in the skin after a few days. The so-called blue pigmentation of *some* morphomaniacs is merely an exaggeration of this, due to constantly repeated injections being each followed by a blue iron mark in the skin.

The large field of comparative medicine is still uncultivated for the most part, and we must therefore be thankful for any research that throws light upon the diseases that are common to man as well as to the lower animals. Of late years special study has been paid to the bone diseases of domesticated animals, and we need only point to Klapp's investigations on scoliosis in animals to show how instructive such observations may be. An interesting contribution to this study has recently been made by Dr. Dräseke, who demonstrated before the Hamburg Medical Society a remarkable pathological specimen taken from the elephant "Anton" that died after thirty-six years of captivity in the local zoological garden. This specimen is a portion of the spinal column of the animal, which shows a partial ankylosis of the dorsal vertebræ. Clinically the animal had shown no signs of such a defect during life, except that its movements had been unusually slow and stately. In the discussion that followed the general opinion was that the ankylosis was partly secondary, and that the condition was due to spondylitis deformans. In animals this disease is not very rare, and has frequently been observed in dogs and horses. In this case, however, it is somewhat difficult to see to what the ankylosis is due, as the weight-pressure theory which is called in to explain the causation of spondylitis deformans in man is hardly applicable here.

---

Best, of Dresden, in a recent article dealing with the prevention of myopia, shows that during the last decade shortsightedness has been steadily on the increase in Germany. In 1866 Cohn found that 60 per cent. of scholars whom he examined were myopic; Gärtner in 1882 found 82 per cent. According to the author it is no exaggeration to say that at least 50 per cent. of all pupils attending German schools are shortsighted. "We Germans," he remarks, "are said to be a nation of myopics, a nation that wears spectacles; but we are also the nation that first carried into practice the principle of compulsory education." Although the percentage of myopics in elementary schools is high, it increases in the advanced classes, and the highest figure is given by the University students. The factors that account for this high percentage are various, but Best declares that the main cause is a disposition to shortsightedness due to congenital, and presumably hereditary, eye defects. That to a certain extent heredity is an important factor is incontestable, but there are many points in the causation of myopia which are as yet obscure and in need of further explanation. After discussing the generally accepted theories of causation, the author concludes that shortsightedness is undoubtedly due to defect of the growing period, and that it is important, therefore, to find out what factors regulate the growth of the eye. He is disposed to lay great stress on the importance of the activity of the eye muscles, and finds that in most cases of myopia there are definite, though slight, structural defects in the eye.

Professor Cramer, of Göttingen, who is well known for his contributions to neurology, discusses in a recent number of the *Munchner Medizinische Wochenschrift* certain questions relative to the reform of statutory legislation from a psychiatrical point of view. This paper is an abstract of an address delivered before the German Psychiatrical Association in April last, and although it concerns itself more with German law, some of the proposals have a general interest. Thus, acknowledging the difficulty that the expert witness often has when he is called upon to express an opinion as to the mental condition of a particular prisoner, he urges the establishment of "observation institutions" in which doubtful or border-line cases can be closely examined and observed. The objections which have been urged against such establishments, which naturally would have to deal only with exceptional cases, he believes to have been exaggerated. Another important point touched upon is the mental capacity of witnesses who have been insane or mentally unsound, but have recovered and are to all intents and purposes cured. At present the judge decides as to the capacity of the witness, but Cramer proposes that in all such cases the psychiatrical expert should be consulted and should give an opinion after closely examining the witness. The judge should, in all cases where there is any question as to the mental soundness or unsoundness of the accused or of any of the witnesses, be able to call to his aid an acknowledged expert, and should be able to differentiate between "the medical man who writes on mental diseases in the lay Press" and the experienced psychiatrist. In order to do so he should be acquainted with psychiatrical literature, so far at least as it concerns medico-legal questions.

---

One of the most troublesome and distressing symptoms of epidemic cerebro-spinal meningitis is the incessant vomiting which often fails to yield to the ordinary methods of treatment of the disease. It is worth while recording, therefore, that Dr. Arnold claims to have obtained complete relief in several cases by the administration of hydrochloric acid several times a day before food. He was induced to try this medicament by observing on analysis that the vomits were always free from hydrochloric acid. It occurred to him, therefore, that its administration might have a favourable influence upon the sickness, and was soon convinced that such indeed was the fact. The vomiting diminished in frequency and in the course of a day or two ceased altogether. At the same time, considerable amelioration took place in the patient's general condition, the appetite returned and the temperature fell. He has found it advantageous also to add a little pepsin.

---

It is frequently urged by fever authorities that in order to ensure success in the treatment of diphtheria with antitoxin the serum must be injected in sufficiently large quantities and at the earliest possible stage of the disease. It is, however, at the same time, generally recognised that no further benefit is obtained by pushing the dose of antitoxin beyond certain limits. Dr. Dronys Pospischil, of Vienna, holds a contrary opinion and has worked out a method of treatment based upon a study of nearly 100 cases. The essential principle of the treatment consists in the administration of enormous doses of the serum amounting to 20 to 30 times 1,500 units. He favours "local" injections at the angle of the lower jaw, and as soon as a case is recognised as serious he injects three or four bottles on each side. Pigmentation of the urine occurs as a result of the carbolic acid in the serum, but this soon disappears. There is also much œdema at the site of injection, but this also subsides within 24 hours. Swellings of the glands, elevation of temperature, and fœtor disappear at once. If there is tachycardia and bronchitis, digitalis and caffeine are recommended, and when the general condition is bad, with intermittent pulse and low tension, the author resorts to hypodermic injections of adrenalin. Dr. Pospischil appears to have much confidence in the efficacy of adrenalin in these grave cases. He administers two to four injections per diem of 3 grammes each, and does not hesitate to give to children even as much as 200 grammes of adrenalin in desperate cases. In order to procure sleep and freedom from restlessness the author falls back upon morphia as the best remedy.

---

Dr. Bernard Fischer, professor of pathology at Bonn, has drawn attention to a serious accident which may happen in the course of an abdominal section, namely, the strangling of a loop of small intestine under a deep through and through suture of the abdominal wall. Dr. Fischer has observed post-mortem three cases of such a retro-parietal incarceration under a suture and he states that in each case the operation was performed by an experienced surgeon. In cases of laparotomy for intestinal obstruction, owing to the distended condition of the bowel there may be difficulty in bringing the edges of abdominal incision together, and recourse is then made to some deep sutures passing right through the abdominal wall. It is between one of these sutures and the abdominal wall that a loop of intestine may get caught and strangled. Such was the condition in all three cases. In each case it was supposed that the patient was suffering from paralytic distension due to infection; but the autopsy revealed the actual condition. But this is not the only mishap that may occur from abdominal suturing. Professor Fischer has also found, post-mortem, that the needle has penetrated a loop of intestine and sutured it to the parietal peritoneum. He thinks that such accidents are overlooked by reason of the custom of opening the abdomen post-mortem through the original incision of the surgeon. He has adopted the method of always making an opening at the side of the original incision and immediately inspecting the internal aspect of the line of sutures. It is in this way that the author has discovered these surgical mishaps. Although such an accident is probably most exceptional, the fact that it has occurred more than once in the experience of one pathologist, who has looked out for it, emphasises the extreme importance of the abdominal suture and the care and attention that should be given to it.

Although the work of the Royal Commission on Tuberculosis has pretty clearly demonstrated the relationship between bovine and human tuberculosis, and has established almost beyond dispute the possibility of human infection from cattle, research has been seriously hampered (as in fact it is in all such cases) by the impossibility of inoculation experiments on the human individual. Dr. F. L. Pochin, of Fakenham, has attempted to circumvent this difficulty by an ingenious use of the opsonic index, and his results are certainly interesting. He obtained emulsions of human and bovine tubercle bacilli from the Lister Institute as nearly as possible of the same strength, and he estimated the opsonic indices of 10 healthy young adult cattle, of 10 children and of ten adult individuals to both strains. In each case he took the average of 100 counts. In the cattle series a much higher opsonic index is shown for the human bacilli than for the bovine, so that on the grand average for 1,000 counts among the 10 cattle about 85 per cent. more human bacilli were taken up by the leucocytes than was the case with the bovine strain. Conversely, in the human series, both for children and adults, the averages show a resistance to the bovine type greater by 254 per cent. than that to the human type of tubercle bacilli. Great as the difference thus appears to be in the resistance of man and cattle to the two strains respectively, Dr. Pochin thinks that the figures rather confirm the findings of the Royal Commission. It can in truth hardly be said that any definite conclusions can be drawn either one way or the other, for it is quite unascertained what degree of opsonic power constitutes an absolute immunity to a given infection. The method, however, offers a means of comparison between the resistances of different species to different strains of micro-organisms and may prove of value also in other lines of bacteriological research.

---

A recent number of *La Revue de Médecine* contains an interesting article by Barié *à propos* of the volume of the heart in patients suffering from chlorosis. The author, in order to determine this volume, examined a number of normal persons of various ages and mapped out upon them by percussion the area of relative cardiac dulness. Care was taken to examine them all fasting and in the position of dorsal decubitus. By careful measurements he was able to get a standard of cardiac volume at each age wherewith to compare results obtained by similar means from chlorotic patients. He found that in eighteen chlorotic patients so examined nine showed a reduction, three an increase of cardiac volume as compared with the normal person of the same age, whereas six showed no departure from the normal. It was known half a century ago that a reduction in cardiac volume is common in this disease. Rokitansky attributed it to congenital atrophy of the heart. Virchow, on the other hand, considered chlorosis to be due to a hypoplasia of the aorta due to arrested development in which the heart joins. The author's researches partly confirm the existence of this atrophy, but they also show that the cardiac volume of chlorotics is not so frequently diminished as has been thought, seeing that in one-third of his cases the volume remained normal, and that it was increased in one-sixth of the cases. Mechel and Beau maintained that dilatation of the heart is frequent in chlorosis, and many theories were advanced to account for this. One of them assumed that the dilatation is apparent and not real and is due to upward displacement of the diaphragm. This displacement has recently been shown to take place by Grunmach, who examined the chlorotic heart by means of radiography; but it is exceptional, and in most cases the dilatation when present is a true one. Gerhardt believed that the dilatation only occurs in the left ventricle, and then often produces a soft murmur heard at the apex.

---

Seeing the frequency of exocardial murmurs in chlorosis, Barié is of opinion that many of these so-called functional murmurs are really of cardio-pulmonary origin. His observations show that the phenomenon of dilatation varies in different cases. In one of his cases the right heart alone was involved, and in some others the heart as a whole. In most cases the dilatation never approaches the severity of that due to an organic lesion. Digestive troubles, which are common among chlorotics, tend to favour dilatation by hindering the proper nourishment of the myocardium. These patients are also of neurotic temperament, frequently suffering from hysteria, neurasthenia, etc., conditions which tend to favour dilatation of the heart reflexly. This reflex dilatation may also result from troubles of other abdominal viscera such as the liver and intestines. As the result of his researches Barié draws the following conclusions: An appreciable reduction of cardiac volume is frequently to be observed in chlorosis. Some circumstances, however, are capable of producing a cardiac dilatation; these are principally myocardial atony, certain gastric dyspepsias, and various neuropathic conditions. Whatever be their origin, these dilatations are usually temporary and slight.

---

But little is known of the pathology of rectal neuralgia, a disease which is more common in the female sex and which can only be diagnosed by exclusion. In the *Deutsche Med. Zeitung*, Dr. Albu calls attention to the symptomatology and treatment of the condition. The symptoms are vague pains in the rectum and anus, radiating into the buttocks, coccyx, perineum, and thighs. The pains may be either sharp and lancinating or may take on the character of lightning pains. Treatment consists in the administration of soothing drugs, either by the rectal or buccal route or subcutaneously. The best drug is perhaps belladonna in strong doses. Opium and morphine should under no circumstances be given. In addition to drug treatment hot hip-baths, vapour douches, and compresses of mineral earths have their uses. Constipation should be guarded against. When the condition of the patient has improved somewhat the introduction of rectal sounds of large calibre often renders the improvement permanent.

